# Use of Invasive Green Crab *Carcinus maenas* for Production of a Fermented Condiment

**DOI:** 10.3390/foods10040659

**Published:** 2021-03-24

**Authors:** Delaney M. Greiner, Denise I. Skonberg, Lewis B. Perkins, Jennifer J. Perry

**Affiliations:** School of Food and Agriculture, The University of Maine, Orono, ME 04469, USA; delaney.greiner@maine.edu (D.M.G.); denise.skonberg@maine.edu (D.I.S.); lewis.perkins@maine.edu (L.B.P.)

**Keywords:** *Carcinus maenas*, histamine, fermentation, fish sauce, green crab

## Abstract

To control the population of an invasive species of green crab, we investigated the feasibility of producing a fermented crab condiment. Commercial fermented fish condiments were tested to assess variability in the marketplace and to identify targets for lab-fermented sauces. Finely chopped crab was combined with 100 mg g^−1^, 200 mg g^−1^, or 300 mg g^−1^ NaCl, and spontaneously fermented for up to 120 days. Chromatographic analysis revealed that histamine content was not a safety concern as all treatments were below the current U.S. legal threshold (50 mg 100 mL^−1^). The majority of microbial and physicochemical properties measured within salt level (proteolytic bacterial population, total volatile basic nitrogen (TVBN), amine nitrogen, water activity, moisture, and biogenic amines) were statistically unchanged between days 60 and 120 of fermentation, suggesting that most of the biochemical changes happened early in the fermentation. While the production of a fermented condiment was successful and could represent an opportunity for the valorization of this invasive species, additional work is needed to accelerate the process and further understand the dynamics of the early fermentation stages.

## 1. Introduction

The European green crab (*Carcinus maenas*) is an extremely aggressive invasive species established on North America’s east and west coasts [1,2]. The green crab preys on commercially relevant clam and mussel species, which has adversely affected coastal regions ecologically and economically. This predation has decreased soft-shelled clam populations on the east coast of the United States by 40%, representing a loss of $22.6 million per year in sales [2]. Adverse effects of this invasive species are not limited to predation. Mature and juvenile green crabs damage important ecosystems by eating eelgrass and digging through the sediment surrounding eelgrass [3]. These eelgrass beds are a critical habitat, serving as nurseries for juvenile fish, providing protection from native predators, and acting as an essential food resource for diverse species including waterfowl [4]. The presence of green crabs also poses a threat to the American lobster population through competition over prey [5].

Unfortunately, hard shelled green crabs are difficult to use in the culinary industry because of difficulties removing meat from this diminutive species. One opportunity to control this species that has been investigated is the establishment of a soft-shell fishery. This fishery could help exert local control over the population of green crab and is considered low barrier to entry due to the use of existing fishing materials and the lack of trapping limits on this species [1]. In Italy, there are mature soft-shell green crabs selling for as much as €51.14 kg^−1^, or about $27 USD lb^−1^ [1]. Other current uses for the crabs include composting and bait [6], but the establishment of a soft-shelled industry represents the highest value opportunity for fishers.

Pre-molt green crabs are easily identified and can be stored live until suitable for sale in a soft-shell market. However, a significant portion of the trapped crabs are not suitable for live storage. Although this excess biomass could be sold as bait or composted, the establishment of an edible use for the excess material would command a higher price for fishers. This additional valorization of excess biomass further incentivizes fishers and makes the system more sustainable [7].

Fermented fish sauce is a clear liquid, brown in color, with a distinct fish flavor [8], which, in its simplest form, consists of the liquid resulting from the spontaneous fermentation of salted whole anchovies and other fish species [9]. The fermentation of a fish sauce typically ranges between 6 and 18 months [8,10], though duration depends on ambient temperature and local preferences. Fermented seafood sauce is historically a widely used condiment in a large number of cultures, particularly those with Asian influence, and is becoming more common in American kitchens [11]. 

Anchovies are commonly used in fish sauce manufacture because of their low value and abundance of protein, with the Peruvian anchovy having up to 20% protein [12]. Although anchovies are the most commonly used substrate, diverse species including tilapia, sardines, silver barb fish, and freshwater crab have been used in fermented condiment production [13,14,15,16,17]. *Carcinus maenas* has a protein content of about 17% [18], which could be fermented as a way to utilize this valuable protein in the culinary industry without the need to separate the shell and meat. Additionally, this process is relatively low input with little capital investment needed, making this product accessible for small-scale processing operations or cottage producers. The purpose of this work was to assess the physicochemical properties of existing commercial fish sauce products to establish baseline values for a novel, fermented seafood condiment, and to assess the feasibility of using whole green crab as the substrate for such a product.

## 2. Materials and Methods

### 2.1. Preparation of Crab

Green crabs were trapped off the coast of Georgetown, Maine, and transported on ice to the University of Maine (Orono, ME, USA). Live crabs were blast frozen (Southeast Cooler, Lithia Springs, CA, USA) for 1 h at −30 °C, then stored at −20 °C until use. Frozen whole crabs were thawed for 36–48 h at 4 °C before being finely chopped in a Kolsch bowl cutter (UltraSource, Kansas City, MO, USA) and combined with uniodized Kosher salt (Morton Salt, Chicago, IL, USA) at 100 mg g^−1^, 200 mg g^−1^, or 300 mg g^−1^ (*w*/*w*). All treatments were prepared separately, in triplicate, and packed into clean, 0.95 L canning jars covered with a double layer of cheesecloth (Pyrm Consumer USA, Spartanburg, SC, USA).

### 2.2. Fermentation and Sampling

Treatments were incubated at 24 °C for 120 days with intermittent sampling occurring at 60, 90, and 120 days. On each sampling occasion one entire jar from each treatment replicate was utilized (no repeated sampling). The sauce was separated from the solid residue for testing by straining through two layers of non-sterile cheesecloth into 250 mL centrifuge tubes. The filtrate was centrifuged in an Avanti J-E Beckman Coulter centrifuge (Brea, CA, USA) (100× *g*, 10 min), and the supernatant was collected for microbial and physicochemical testing.

### 2.3. Determination of Microbial Activity

Crab sauce was serially diluted in 1 g L^−1^ peptone (Becton, Dickinson, and Co., Sparks, MD, USA) and spread plated in duplicate on skim milk agar (SMA), which consisted of brain heart infusion agar (Hardy Diagnostics, Santa Maria, CA, USA). It was supplemented with 100 mL L^−1^ (*v*/*v*) aseptically packaged skim milk (Natrel, Quebec, Canada) and 30 mg g^−1^ salt (Aqua Solutions, Deer Park, TX, USA; incubated at 37 °C for 48 h) to identify proteolytic bacteria [19]. The brain heart infusion agar was supplemented with 30 mg g^−1^ salt (incubated at 37 °C for 48 h) for total plate count (TPC), and potato dextrose agar (APDA; Alpha Biosciences, Baltimore, MD, USA) was acidified with 0.1 M tartaric acid (Alfa Aesar, Ward Hill, MA, USA; incubated at ambient temperature for 5 day) to isolate fungi. All plates were counted with colony density between 30 and 300, and the microbial population was expressed as log CFU g^−1^.

### 2.4. Determination of Total Volatile Basic Nitrogen (TVBN) and Amine Nitrogen

Total volatile basic nitrogen was measured [20] via direct distillation with sodium hydroxide. Lab fermented crab sauce sample was homogenized with trichloroacetic acid (Sigma-Aldrich, St. Louis, MO, USA) and centrifuged (1312× *g* 20 min). The supernatant was distilled in a micro-Kjeldahl apparatus (Labconco, Kansas City, MO, USA) with sodium hydroxide (Fisher Chemical, Fair Lawn, NJ, USA) and antifoaming agent A (Sigma Aldrich, St. Louis, MO, USA). The distillate was collected in boric acid solution (JT Baker, Center Valley, PA, USA) containing methyl-red (Fisher Scientific, Waltham, MA, USA), a methylene-blue (Sigma Aldrich, St. Louis, MO, USA) indicator, and titrated for a color change with 0.1 N hydrochloric acid (Fisher Scientific, Waltham, MA, USA). TVBN content was expressed as mg 100 mL^−1^.

Proteolytic activity drives increased amine nitrogen content [21]. This measurement is typically used to determine the degree of proteolysis due to activity of endogenous and microbial proteases and can be used to estimate the progress of fermentation. The amine nitrogen was determined using a formol titration published by [22], with the following slight modifications. A concentration of 0.1 N sodium hydroxide (NaOH) was used for the neutralization of the original sample and formaldehyde. Moreover, 8 mL of formaldehyde was added (Fisher Scientific, Waltham, MA, USA). The titrant used for the mixture of neutralized formaldehyde and neutralized crab sauce sample was 0.05 N NaOH. Data were expressed as mgN 100 mL^−1^. 

### 2.5. Determination of pH, Water Activity, and Moisture Content

The pH (Orion Star A111 pH meter, Thermo Scientific, Waltham, MA, USA) was determined through a single, direct reading from each sample. The probe was first calibrated with pH 4, 7, and 10 standards. The water activity values (Aqualab, Pullman, WA, USA) were determined through two direct readings and averaged. The water activity meter was first calibrated with a 0.76 calibration standard. Moisture content (%) was determined using AOAC Method 934.01 [23] at a vacuum of 20 in Hg. 

### 2.6. Determination of Non-Enzymatic Browning

Non-enzymatic browning was measured according to the method of [24] with slight modification. One mL of sauce was stirred using a magnetic stir bar and plate with 10 mL of ethanol (500 mL L^−1^ *v/v* Fisher Scientific, Waltham, MA, USA) for one hour. The mixture was then filtered through a 0.45 μm syringe filter (MDI Membrane, Harrisburg, PA, USA) and subjected to an absorbance measurement at 420 nm with a DU 530 spectrophotometer (Beckman Coulter, Brea, CA, USA). 

### 2.7. Determination of Biogenic Amine Content

Biogenic amines in samples and analytical standards were determined using high performance liquid chromatography (HPLC) and the Waters AccQ-Fluor^TM^ fluorescent tagging system (Milford, MA, USA), a method developed for the determination of amine compounds in foods. The Agilent model 1100/1200 HPLC system included a quaternary pump, autosampler, column heater, fluorescence detector, and Chemstation™ software. Approximately 1 mL of crap sauce was filtered through a 0.45 μM nylon syringe filter (Cole-Palmer, Vernon Hills, IL, USA). Ten μL of sample filtrates and 10 μL of standards were prepared for HPLC analysis with the AccQ·Fluor^TM^ kit and assayed using the HPLC column and eluents supplied with the kit. All procedures included in the kit directions insert were followed, with a slight modification of the HPLC gradient elution. Standard curves were constructed using five concentrations of histamine, agmatine, putrescine, cadaverine, and tyramine (all from Sigma-Aldrich, St. Louis, MO, USA), ranging from 0.557–0.894 mg mL^−1^ and diluted with HPLC-grade water. Baseline separation of the target analytes was achieved and biogenic amines were identified by comparing retention times from samples with the analytical standards. Peak areas were used to calculate analyte concentrations. Data were expressed as mg 100 mL^−1^.

### 2.8. Commercial Fish Sauces

In addition to laboratory-fermented crab sauce, 12 varieties of commercial fish sauce were obtained from local and online retail outlets. These sauces were subjected to the same analyses described above to create a standard for comparison since fermented crab sauce is not commercially available. Commercial sauces were separated into two tiers (Table 1) according to ingredients (Tier 1 comprised of minimal ingredients suggesting traditionally fermented, higher value product, Tier 2 comprised of sauces containing additional ingredients such as colorants and preservatives) and were compared to each other statistically to identify significant differences. Tier 1 samples, on the basis of higher quality, were identified as better targets for laboratory-fermented sauce and were subsequently compared statistically to day 120 prototypes. 

### 2.9. Statistical Analysis

The data were tested for normality using a Shapiro-Wilks test. Outliers were identified and removed when appropriate. The results were analyzed for variance using multivariate ANOVA for normal data and Kruskal–Wallis test for non-normal data. Tukey’s honestly significant difference (HSD) post hoc test was used to identify statistically significant (*p* < 0.05) differences among treatments after testing for variance in R Version 3.6.1 (R Studio, Boston, MA, USA).

## 3. Results

### 3.1. Commercial Fish Sauces

In comparing the ingredients of commercial sauces currently available in the marketplace, two distinct groups emerged. One of these (Tier 1) contained only minimal ingredients characteristic of a traditionally fermented fish sauce, while a second (Tier 2) contained various additional, “non-traditional” ingredients. In order to allow for the calculation of mean values for comparison to lab-fermented sauce, the commercial sauces were separated into two categories (Table 1).

Between the two tiers of commercial product, there were apparent differences in water activity, TVBN, and amine nitrogen. These differences were statistically significant and suggest that the use of traditional vs. non-traditional ingredients results in a different sauce. Tier 2 sauces had a significantly higher water activity, and significantly lower TVBN and amine nitrogen contents than tier 1 sauces. 

The commercial sauces made from traditional ingredients—i.e., fish, salt, and sometimes sugar and water—were selected as a more appropriate target for the experimental product. They were compared to the lab-fermented crab sauces to identify properties of significant difference. The lab fermented crab samples had a significantly higher pH at all salt levels. The water activity of the 100 mg g^−1^ salt lab-fermented sample (0.857 ± 0.004) was also significantly higher than the mean of the tier 1 commercial samples (0.74 ± 0.01), but no difference between higher salt and commercial formulations was detected.

### 3.2. Microbial Activity

Yeast and mold levels in general (Table 2) were low in all treatments and did not have any significant differences due to treatment or time during the fermentation. 

**Figure 1:**
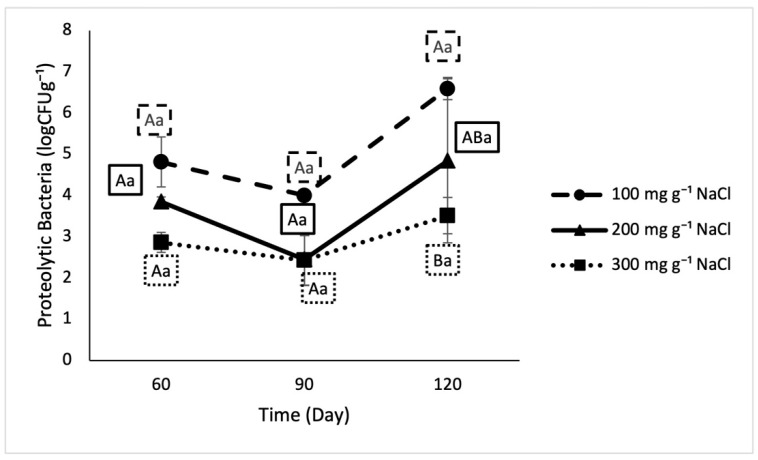
Proteolytic bacterial population in lab fermented crab sauce samples over time. Uppercase letters designate significant differences among treatments on individual testing dates. Lowercase letters designate significant differences within treatment across time (*n* = 3).

No distinct trends were found in the levels of any of the microorganisms of interest (total count, proteolytic bacteria, fungi). Based on the model level effects, an increase in salt content negatively affected the proteolytic bacteria (Figure 1) population. However, the only significant difference among treatments was between 100 mg g^−1^ and 300 mg g^−1^ salt samples (6.6 ± 0.3 logCFU g^−1^ and 3.5 ± 0.4 logCFU g^−1^ respectively) on day 120.

**Figure 2:**
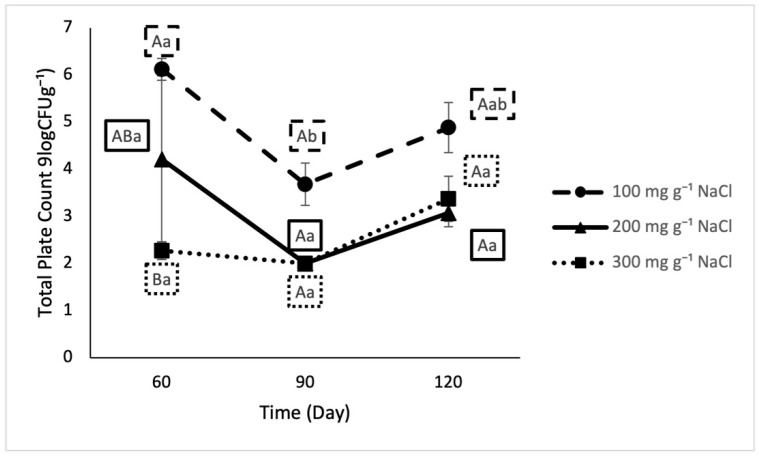
Total plate count in lab fermented crab sauce samples over time. Uppercase letters designate significant differences among treatments on individual testing dates. Lowercase letters designate significant differences within treatment across time (*n* = 3).

Both longer fermentation time and higher salt content had significant model level effects on TPC (Figure 2) that resulted in increased plate count. 

### 3.3. TVBN and Amine N

**Figure 3:**
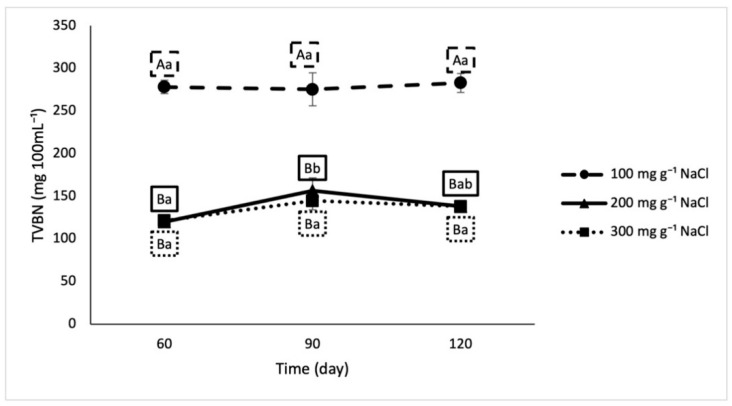
Total volatile basic nitrogen in lab fermented crab sauce samples over time. Uppercase letters designate significant differences between treatments on individual testing dates. Lowercase letters designate significant differences within treatment across time (*n* = 3).

In our analysis, TVBN did not increase over time, but crab sauce formulated with 100 mg g^−1^ salt had significantly higher levels of TVBN (287.9 ± 14.0 mg 100 mL^−1^) than the 200 mg g^−1^ and 300 mg g^−1^ samples (138.2 ± 17.6 and 134.8 ± 11.7 mg 100 mL^−1^, respectively) at all time points (Figure 3). The 200 mg g^−1^ and 300 mg g^−1^ salt treatments were statistically indistinguishable from each other regardless of time.

Although there were significant differences in amine nitrogen levels among treatments, with the 100 mg g^−1^ salt treatment having significantly higher amine nitrogen (907.67 ± 47.72 mg N 100 mL^−1^) than the 200 mg g^−1^ and 300 mg g^−1^ samples (628.06 ± 33.44 and 581.00 ± 39.92 mgN 100 mL^−1^, respectively), the amine nitrogen levels did not increase over time in any treatment (Table 3).

### 3.4. pH, Water Activity, and Moisture Content

**Figure 4:**
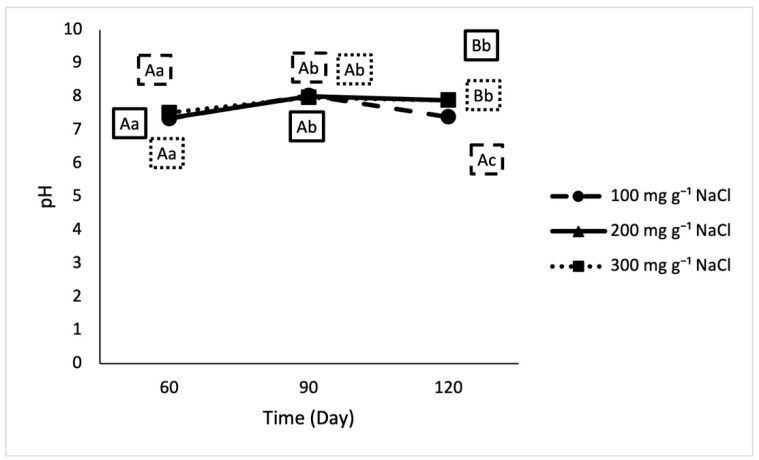
pH in lab fermented crab sauce samples over time. Uppercase letters designate significant differences between treatments on individual testing dates. Lowercase letters designate significant differences within treatment across time (*n* = 3).

Differences in pH were driven by time (Figure 4). From day 90 to 120, the pH of the higher salt samples did not change, but the mean pH of the 100 mg g^−1^ salt sample significantly decreased. 

Crab sauces made with 200 mg g^−1^ (0.748 ± 0.005) and 300 mg g^−1^ (0.744 ± 0.004) salt had significantly (*p* < 0.05) lower water activities than those prepared with 100 mg g^−1^ salt (0.861 ± 0.006) at all time points (Table 4), as expected. 

Similar to water activity, the moisture content (Table 5) of samples made with 200 mg g^−1^ (66.61 ± 2.13%) and 300 mg g^−1^ of salt (68.32 ± 0.43) was significantly lower than those prepared with 100 mg g^−1^ salt (74.52 ± 0.95%) at all time points. 

### 3.5. Non-Enzymatic Browning

**Figure 5:**
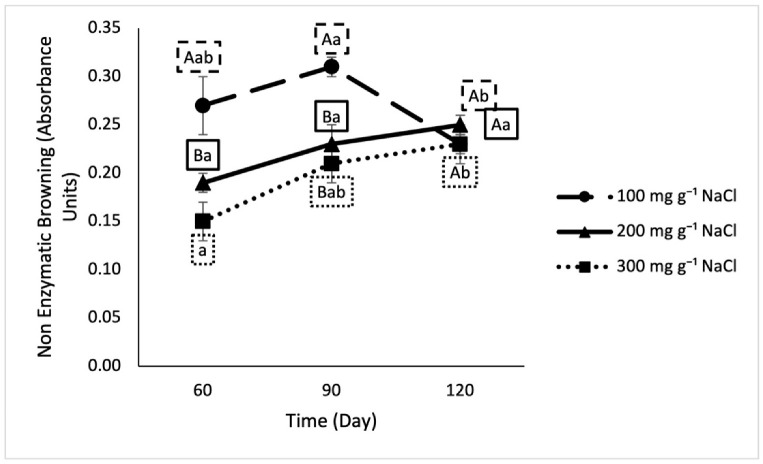
Non-enzymatic browning in lab fermented crab sauce samples over time. Uppercase letters designate significant differences between treatments on individual testing dates. Lowercase letters designate significant differences within treatment across time (*n* = 3).

Non-enzymatic browning is the development of the brown color of the sauce caused by reactions such as the Maillard reaction. The Maillard reaction primarily involves the reaction of reducing sugars, oxidation products, and free amino acids [8]. The degree of non-enzymatic browning (Figure 5) of samples fermented with 200 mg g^−1^ and 300 mg g^−1^ salt was significantly higher than that observed in the 100 mg g^−1^ samples on days 60 and 90, but results were not significantly different due to treatment on day 120. The non-enzymatic browning on day 120 (0.237 ± 0.00) was found not to be significantly different from the Tier 1 commercial samples at any of the salt levels. 

### 3.6. Biogenic Amines

There was no significant effect on the biogenic amine concentration (Table 6) from time or salinity. Data are expressed as averages among time and salinity. Cadaverine and tyramine were not identified in any of the crab sauces. 

## 4. Discussion

Fish sauce is traditionally made through spontaneous fermentation. During spontaneous fish sauce fermentation, proteolytic enzymes break down fish flesh. This proteolysis is induced by endogenous proteases found in the muscles and the digestive tracts of the fish/crab and the activity of natural microbiota. The speed at which this degradation occurs depends on a number of factors including the amount of salt in the formulation and the incubation temperature of the raw materials. The catabolism of proteins yields a varied assortment of nitrogenous compounds responsible for the aroma and flavor of traditional fish sauce [25]. This spontaneous fermentation was the method used to make a fermented condiment from *Carcinus maenas* in this study. The formulations studied consisted of crab and 100 mg g^−1^, 200 mg g^−1^, or 300 mg g^−1^ salt. 

In traditional fish sauce fermentation, proteolytic bacteria are expected to be present at populations of almost 4.0 logCFUg^−1^ after 4 months [19,26]. The proteolytic bacterial population in this study ranged from 2.4–3.5 logCFU g^−1^ after 120 days of fermentation. As the fermentation progresses, the enzymes produced by these bacteria catabolize the proteins in the raw material, resulting in the formation of peptides and amino acids, measured in this study as amine nitrogen. Total volatile base nitrogen also increases with time, giving rise to odiferous compounds that drive the use of this metric as an indicator of spoilage in fish [27]. Because they result from the breakdown of proteins, which characterizes the process of fish sauce fermentation, both of these indices could be expected to increase with increasing fermentation duration (to almost 2400 mg 100 mL^−1^ and 600 mg 100 mL^−1^, respectively) [19,28]. That was not, however, observed in our study. Data in this study suggests that the majority of protein catabolism occurred in the very early stages of the fermentation.

In some instances, depending on the microbiota present in the system, amino acids are decarboxylated to release biogenic amines, such as histamine. Biogenic amine content can be an indicator of product quality and aids in determining acceptability of a commercial sample. A high histamine content specifically can cause scombroid poisoning, which causes rash, swelling, and vomiting [28]. As a result, in the United States, fish sauce cannot legally contain more than 50 mg 100 mL^−1^ of histamine [29]. Histamine levels were low in experimental product, which could suggest that the native flora of *Carcinus maenas* is largely non-histamine producing. Agmatine was not recovered from most commercial samples but was frequently identified in lab fermented sauces, likely due to the difference of arginine content in the starting material. None of the lab fermented samples or commercial samples had a histamine concentration above the legal limit. 

Water in foods drives a number of degradative processes. Water activity measures the amount of free water available for bacterial growth [30]. Most pathogenic bacteria are unable to grow at a water activity below 0.9 [31], indicating that vegetative pathogens are unlikely to pose significant food safety concerns for this fermented crab product, particularly in the higher salt formulations, which both had water activity values well below 0.8. 

There is very little information in the literature regarding the sensory aspects driving acceptability of fermented fish sauces. As a result of this fact, we chose to complete a small scale survey of commercial products in order to orient the experimental crab sauce in comparison to potential physicochemical targets. The resulting fermented crab condiment looked and smelled similar to the commercial fish sauces in that it was a dark brown color and smelled like fermented seafood. The pH was higher in all lab samples than the commercially available samples, most likely due to the higher levels of lactic acid bacterial population associated with fish sauce fermentation [17]. There were no differences between the lab and commercial samples in amine nitrogen content, which shows that the degree of proteolysis that occurred in the lab samples was comparable to the commercial samples. Comparable levels of browning suggest that the color of the experimental crab sauce has the potential to meet current consumer expectations. Levels of TVBN and moisture (in all salt formulas) were also similar to commercial product. However, better characterization of the first 60 days of fermentation is warranted. Likewise, consumer acceptability testing is needed in order to assure the palatability of a green crab sauce. Additional investigation should also assess whether the process could be accelerated or consumer appeal could be increased by modification of formula or process alterations.

## 5. Conclusions

The concept of creating a fermented condiment from *Carcinus maenas* was proven to be a feasible opportunity. The experimental crab sauce product was comparable in many respects to currently available commercial fish sauce products. The optimization of the product and process, as well as consumer sensory evaluation, are called for to ensure the market potential of such a product. The establishment of an economically feasible use for green crabs unsuitable for the high value softshell market will be a key accomplishment in facilitating the development of a viable U.S. fishery for this invasive species and realizing the associated ecological benefits of controlling its population. 

## Figures and Tables

**Table 1:** Commercial fish sauce physicochemical characteristics.

	Tier 1 ^a^ (*n* = 5)	Tier 2 ^b^ (*n* = 7)
Ingredients ^c^	anchovy fish, sea salt, sugar, water	anchovy extract, water, salt, mackerel extract, potassium sorbate, fermented scad fish extract, caramel color, syrup, sugar
pH	5.56 ± 0.29	5.41 ± 0.58
Water Activity	0.739 ± 0.014 *	0.774 ± 0.027 *
TVBN (mgN 100 mL^−1^)	390.5 ± 129.4 *	138.3 ± 141.9 *
AmineN (mgN 100 mL^−1^)	1271.2 ± 528.8 *	449.0 ± 447.8 *
Moisture (%)	59.3 ± 4.1	65.9 ± 6.8
Browning	0.53 ± 0.24	0.29 ± 0.29
Histamine (mg 100 mL^−1^)	3.49 ± 1.69	1.82 ± 0.61
Putrescine (mg 100 mL^−1^)	3.93 ± 2.84	3.50 ± 3.62
Cadaverine (mg 100 mL^−1^)	0.81 ± 0.24	0.75 ± 0.42
Tyramine (mg 100 mL^−1^)	2.09 ± 0.41	1.46 ± 0.81

Data are expressed as mean ± standard deviation. * indicates significant differences in mean values between tiers. ^a^ Includes the following brands: A Taste of Thai, Four Elephants, Son Sauce, Red Boat, Golden Boy. ^b^ Includes the following brands: Nuoc Mam Nhi, Purfina Patis, Tentay Patis, Lucky, Mega Chef, Essential, Squid Brand. ^c^ Individual sauce ingredients comprise various combinations of listed ingredients within tier.

**Table 2:** Fungal population in lab fermented crab sauces.

Time (Day)	100 mg g^−1^ Salt (logCFUg^−1^)	200 mg g^−1^ Salt (logCFUg^−1^)	300 mg g^−1^ Salt (logCFUg^−1^)
60	2.5 ± 0.7	2.7 ± 0.5	2.5 ± 0.8
90	2.1 ± 0.1	2.0 ± 0.0	2.0 ± 0.0
120	2.2 ± 0.1	2.2 ± 0.2	2.5 ± 0.4

Data are expressed as mean ± standard deviation (*n* = 3), no statistical differences identified.

**Table 3:** Amine nitrogen in lab fermented crab sauce samples.

Time (Day)	100 mg g^−1^ Salt (mgN 100 mL^−1^)	200 mg g^−1^ Salt (mgN 100 mL^−1^)	300 mg g^−1^ Salt (mgN 100 mL^−1^)
60	884.3 ± 56.5 _a_	581.0 ± 17.4 _b_	539.0 ± 9.9 _b_
90	864.5 ± 168.5 _a_	655.7 ± 1.6 _b_	569.33 ± 46.2 _b_
120	974.2 ± 24.6 _a_	647.5 ± 32.2 _b_	634.7 ± 10.0 _b_

Lowercase letters designate significant differences between treatments on individual testing dates. Data are expressed as mean ± standard deviation (*n* = 3).

**Table 4:** Water activity in lab fermented crab sauce samples.

Time (Day)	100 mg g^−1^ Salt	200 mg g^−1^ Salt	300 mg g^−1^ Salt
60	0.866 ± 0.004 _a_	0.744 ± 0.001 _b_	0.741 ± 0.001 _b_
90	0.862 ± 0.005 _a_	0.751 ± 0.007 _b_	0.744 ± 0.003 _b_
120	0.857 ± 0.004 _a_	0.749 ± 0.002 _b_	0.748 ± 0.002 _b_

Lowercase letters designate significant differences between treatments on individual testing dates. Data are expressed as mean ± standard deviation (*n* = 3).

**Table 5:** Moisture in lab fermented crab sauce samples.

Time (Day)	100 mg g^−1^ Salt (%)	200 mg g^−1^ Salt (%)	300 mg g^−1^ Salt (%)
60	75.8 ± 0.4 _a_	67.5 ± 0.3 _b_	68.6 ± 0.6 _b_
90	73.8 ± 2.8 _a_	68.7 ± 0.7 _b_	68.7 ± 2.8 _b_
120	73.9 ± 1.9 _a_	63.7 ± 9.0 _b_	67.7 ± 0.4 _b_

Lowercase letters designate significant differences between treatments on individual testing dates. Data are expressed as mean ± standard deviation (*n* = 3).

**Table 6:** Biogenic Amine Concentration of Lab Fermented Crab Sauce Samples.

Biogenic Amine	Average Concentration (mg 100 mL^−1^)
Histamine	6.01 ± 1.54
Putrescine	3.50 ± 1.69
Agmatine	4.34 ± 5.07

Data are expressed as mean ± standard deviation (*n* = 27), not statistical differences detected.

## Data Availability

Data available upon request from corresponding author.
